# Prenatal case report of Barth syndrome caused by novel *TAFAZZIN* mutation: Clinical characteristics of fetal dilated cardiomyopathy with ascites

**DOI:** 10.3389/fped.2022.1004485

**Published:** 2022-11-09

**Authors:** Xuliang Zhao, Xu Li, Weiwei Sun, Jian-an Jia, Min Yu, Ruixia Tian

**Affiliations:** ^1^ Department of Laboratory, The 901th Hospital of the Joint Service of the People's Liberation Army, Hefei, China; ^2^ Department of Radiology, Anhui Children's Hospital, Hefei, China; ^3^ Department of Medical, Beijing Chigene Translational Medicine Research Center, Beijing, China; ^4^ Department of Obstetrics and Gynecology, The 901th Hospital of the Joint Service of the People's Liberation Army, Hefei, China

**Keywords:** tafazzin, prenatal diagnosis, LVNC, WES, congenital heart disease

## Abstract

Barth syndrome (BTHS) is a rare X-linked recessive genetic disease, which appears in infancy with myocardial and skeletal muscle diseases, neutropenia, growth retardation, and other clinical features. *TAFAZZIN* is the pathogenic gene of BTHS, which encodes the tafazzin protein of the inner membrane of the mitochondria, a phosphatidyltransferase involved in cardiolipin remodeling and functional maturation. At present, BTHS has been widely reported, but prenatal cases are rare. We report a 24^+4^-week fetus with clinical manifestations including left ventricular insufficiency and ascites. After induced labor, whole exome sequencing detection of fetal skin tissue showed that *TAFAZZIN* had the mutation c.311A > C/*p*.His104Pro and that his mother was the carrier. This His104Pro mutation has hitherto not been reported, and it is rated as likely to be pathogenic according to the American College of Medical Genetics and Genetics guidelines. Molecular dynamics and protein expression experiments on the His104Pro mutation showed that the stability of the local protein structure and protein expression were reduced. In conclusion, the case presented in this study enriches our knowledge of the *TAFAZZIN* mutation spectrum and suggests that His104Pro may lead to cardiac structural abnormalities in the early embryo. The possibility of BTHS should be considered when an abnormal cardiac structure or ascites appear in a prenatal ultrasound.

## Introduction

Barth syndrome (BTHS, OMIM#302060) is a rare X-linked recessive disorder characterized by cardiomyopathy, skeletal myopathy, growth retardation, neutropenia, and increased urinary excretion of 3-methylglutaric acid (3-MGCA) (1). This syndrome was first reported in 1983 and described as a triad of mitochondrial myopathy, neutropenia, and dilated cardiomyopathy (DCM) with high mortality in infants (2, 3). *TAFAZZIN* mutation is the main causative factor of BTHS. This gene encodes the tafazzin (TAFAZZIN) protein, a phosphatidyltransferase located in the inner membrane of the mitochondria, which plays a key role in cardiolipin (CL) remodeling. Decreased TAFAZZIN enzyme activity can affect the formation of respiratory chain supercomplexes and cause cardiomyopathy (4, 5). Children with BTHS mainly present with DCM, and more than half of the cases are accompanied by left ventricular non-compaction (LVNC) (1, 5). Although the cardiomyopathy phenotype of patients will gradually improve over time or remain stable after a certain point, their cardiac ejection function may continue to decrease. Thus, heart failure may be a major cause of death in patients with BTHS (3, 5).

To date, BTHS has been widely reported, but prenatal cases are relatively rare. Here, we identified a fetus with an abnormal cardiac structure, cardiac insufficiency, and ascites upon prenatal ultrasound examination at only 24^+4^ weeks. After induced labor, the skin tissue was tested for genetic diagnosis by trio-whole exome sequencing (trio-WES). Bioinformatic prediction and construction of a molecular dynamics (MD) model showed that a novel *TAFAZZIN* missense mutation was the key pathogenic factor for the fetal phenotype in this case. The results of this study enrich our knowledge of the mutation spectrum of *TAFAZZIN* and provide a reliable basis for predicting recurrence risk in future pregnancies for the genetic counseling of the family.

## Case report

### Case presentation

**Figure 1:**
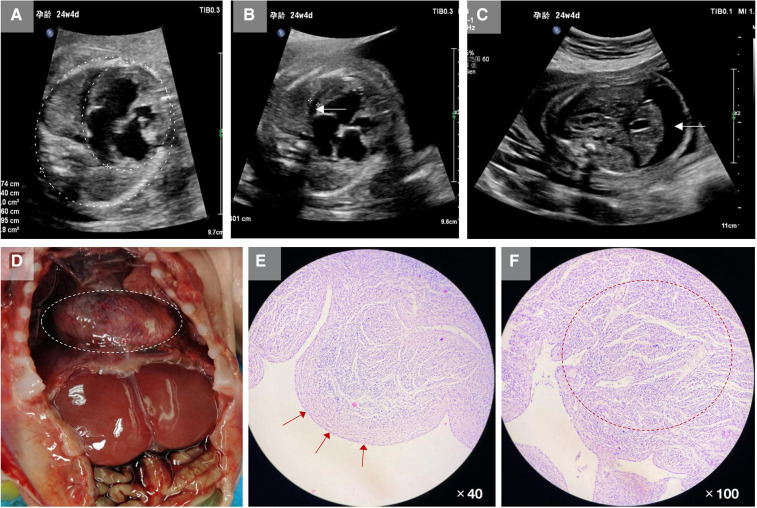
Clinical characteristics of the fetus in this study. (**A**) Ultrasound showed that the fetal cardiothoracic area ratio was increased, approximately 0.45, and the left atrium and left ventricle sizes were increased substantially. (**B**) Ultrasound showed hypertrophy of fetal myocardium and free fluid in the pericardial cavity (white arrow). (**C**) Ultrasound showed a large amount of free fluid in the fetal abdominal cavity and thickened abdominal skin tissue (white arrow). (**D**) After induced labor, autopsy revealed the heart was considerably enlarged. (**E**) Pathological sections showed thickening and fibrosis of the endocardial tissue (red arrows). (**F**) The muscular trabeculae were thick, the sinus recess of the myocardium persisted, the deep depression was staggered, the formation of dense myocardium in the corresponding area was reduced and thin, and the ventricular wall muscle layer remained loose (red circle).

A 31-year-old pregnant woman presented to us, mentioning that her first fetus was induced following fetal heart abnormalities and ascites. Routine ultrasound examination at 24^+4^ weeks of this pregnancy showed that the biparietal diameter of the fetus was 57 mm, the head circumference was 218 mm, and the femoral length was 45 mm. In the fetal heart, the apex of the heart pointed to the left side of the chest, and the cardiothoracic proportion was high, at approximately 0.45. The left atrium and ventricle were obviously enlarged, and the oval valve of the atrial septum was visible. Myocardial thickening was observed. The left ventricular wall was approximately 4.2 mm thick, while the right was approximately 2.2 mm thick. Free fluid was visible in the pericardial cavity, with approximately 4.0 and 3.7 mm outside the left and right ventricles, respectively. We noted pulmonary artery stenosis, pulmonary valve echo enhancement and thickening, and no obvious opening and closing movement. Color Doppler flow imaging showed moderate regurgitation at the mitral valve orifice of the fetus, with a regurgitation velocity of 210 cm/s. Full systolic regurgitation was visible at the tricuspid valve orifice, and the regurgitation velocity was 180 cm/s. A wave reversal was visible in the venous catheter, and pulsation was visible in the umbilical vein. In addition, 16 mm free fluid and thickening of abdominal subcutaneous tissue were seen in the abdominal cavity of the fetus (Figures 1A–C).

Both parents are healthy people in a non-consanguineous marriage. After being fully informed of the relevant risks, the couple decided to terminate their pregnancy. After induction of labor, physical examination showed that the fetal abdomen was swollen. The autopsy revealed increased fetal ascites, a significantly enlarged heart, and hypertrophy of the myocardium. The fetal heart tissue was taken for pathological examination, which showed that the endocardium was thickened and fibrotic, the dense myocardium was relatively thin, and the ventricular wall muscle layer remained loose (Figures 1D–F).

### Genetic analysis

The work described in this case report was conducted in accordance with the Code of Ethics of the World Medical Association (Declaration of Helsinki) for experiments involving humans (https://www.wma.net/policies-post/wma-declaration-of-helsinki-ethical-principles-for-medical-research-involving-human-subjects/). This study was reviewed by the ethics committee of the 901st Hospital of the Joint Service of the People's Liberation Army (ID: 202112001). The pregnant woman and her family members signed informed consent statements for this study and agreed to the publication of the clinical data and images of her fetus.

We collected the induced labor fetal tissue (approximately 2 g of skin tissue from the inner thigh) and extracted 3 ml of peripheral venous blood (treated with EDTA for anticoagulation) from both husband and wife for trio-WES. Leukocyte DNA was extracted according to the operation steps of the genome extraction kit (CoWin Biotech Co., Inc., Beijing, China). After the library was constructed, the designed sequence was captured by an Illumina NovaSeq 6,000 high-throughput sequencer (Illumina Co., Inc., San Diego, CA, USA). The screened mutations were checked against frequency databases of normal people, including dbSNP (www.ncbi.nlm.nih.gov/snp/), ExAC (www.exac.broadinstitute.org/), and 1,000 Genomes (www.1000genomes.org/), and the hazard was predicted and analyzed.

**Figure 2:**
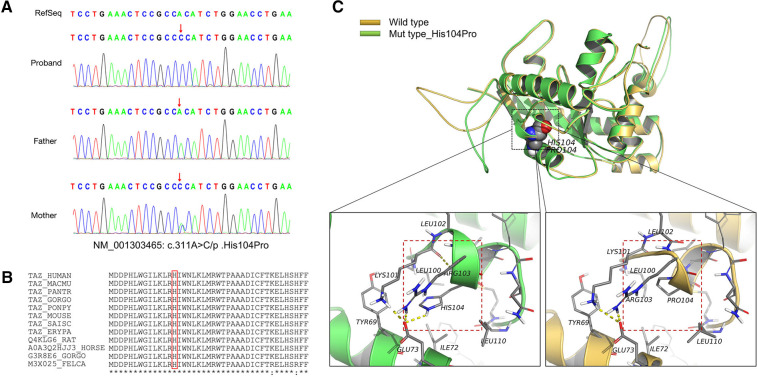
Information on the mutation found in this case. (**A**) The results of gene detection showed that *TAFAZZIN* had a missense mutation, c.311A > C/*p*.His104Pro. The father carried the wild-type form, while the mother carried the mutation. Sanger sequencing confirmed the existence of the mutation. (**B**) His104 is conserved between species (red box). (**C**) The crystal structure model analysis results show that the main chain N–H bond of His104 forms a hydrogen bond with Lys101, and the side chain forms a hydrogen bond with Glu73. When the mutation of His104Pro occurs, the hydrogen bond between Lys101 and Glu73 is broken because Pro104 has a five-membered ring structure with greater rigid strength than the straight-chain structure. Simultaneously, owing to the steric hindrance of Pro104, the secondary structure (part of the *α*-helix) may become a loop domain (red box). Therefore, the His104Pro mutation may reduce the stability of the local protein structure and affect its biological function.

The genetic sequencing results showed that the mutation occurred in exon 3 of *TAFAZZIN* on the fetal hemizygote, NM_001303465: c.311A > C/*p*.His104Pro. The father had the wild-type form, and the mother carried the mutation. Sanger sequencing confirmed the existence of the mutation (Figure 2A). This mutation was not included in the frequency database of normal people, and biohazard prediction and analysis software suggested that this variation can lead to changes in protein structure (Table 1). According to the guidelines of the American College of Medical Genetics and Genomics, the His104Pro mutation is rated as likely to be pathogenic, and the rating evidence was PM1 + PM2 + PP3 + PP4 (6).

**Table 1:** Hazard prediction and analysis of the novel *TAFAZZIN* mutation.

Gene	*TAFAZZIN* (OMIM#300394)
Transcript	NM_001303465
Variant	c.311A > C/*p*.His104Pro
*Prediction software*	
Provean	Deleterious (−5.1)
SIFT (www.sift.bii.a-star.edu.sg/)	Damaging (0.039)
Polyphen2_HDIV (www.sift.bii.a-star.edu.sg/)	Benign (0.0)
Polyphen2_HVAR (www.sift.bii.a-star.edu.sg/)	Benign (0.001)
MutationTaster (www.mutationtaster.org/)	Disease_causing (0.999954)
M-CAP (http://bejerano.stanford.edu/mcap/)	Damaging (0.763338)
REVEL	Deleterious (0.707)
*Distributed frequency database*	
dbSNP (www.ncbi.nlm.nih.gov/snp/)	Not included
ExAC (www.exac.broadinstitute.org/)	Not included
1,000 Genome (www.1000genomes.org/)	Not included

### MD analysis

In brief, we queried the TAFAZZIN protein sequence in the National Center for Biotechnology Information database (https://www.ncbi.nlm.nih.gov/nuccore/NM_001303465.2/) and downloaded the crystal model file of TAFAZZIN from the UniProt database (https://www.uniprot.org/uniprotkb/Q16635/entry#structure). We used single template modeling in Modeller10.1 (https://salilab.org/modeller/). The His104Pro mutant structure was constructed using PyMOL 2.5 (https://pymol.org/2/), based on the crystal structure, and wild-type and mutant proteins were simulated by MD in the GROMACS 5.1.4 program package (http://www.gromacs.org/) under a constant pressure and temperature ensemble. Finally, Chimera 1.15 (http://www.cgl.ucsf.edu/chimera/) was used to analyze the interaction.

MD results showed that the wild-type and mutant structures reached equilibrium at approximately 4,000 ps, and the value after equilibrium was approximately 2.6 Å. Conservation analysis showed that His104 was highly conserved among various species. The results of structural analysis showed that the main chain of wild-type His104 formed a hydrogen bond with Lys101, and the side chain formed a hydrogen bond with Glu73. The His104Pro mutation breaks the hydrogen bond between Lys101 and Glu73. In addition, because Pro104 is a five-membered ring containing an N structure with strong structural rigidity, the spatial steric resistance formed may cause the structure of the *α*-helix to become a loop, affecting its ability to interact with surrounding structures and resulting in reduced local structural stability (Figures 2B,C).

### Western blot (WB)

**Figure 3:**
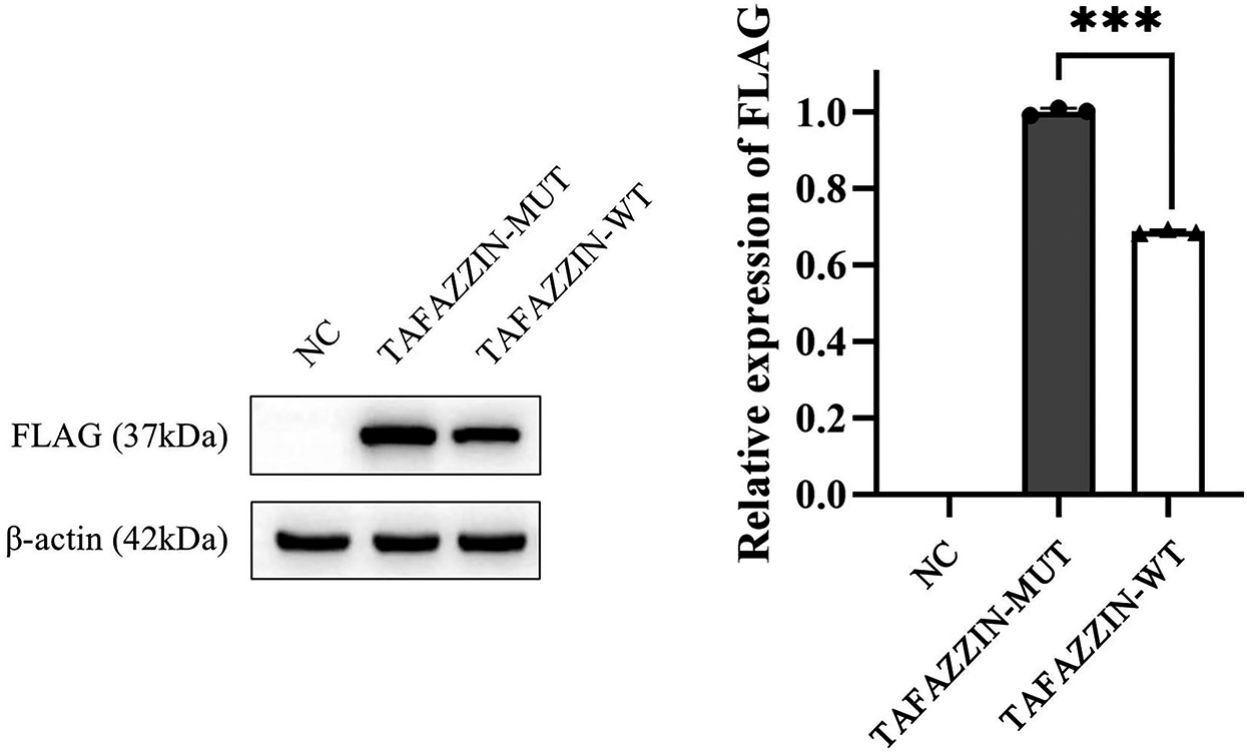
Western blotting results show that the protein expression in TAFAZZIN-MUT (the His104Pro mutant) is significantly lower than that in TAFAZZIN-WT (the wild type), indicating that His104Pro may reduce human TAFAZZIN protein expression and affect relevant biological functions. *, *p* < 0.05; NC, negative control.

We first constructed wild-type (TAFAZZIN-WT) and mutant (TAFAZZIN-MUT) plasmids of TAFAZZIN: the target fragment of TAFAZZIN-WT was amplified with high fidelity polymerase Phanta® Max Super-Fidelity DNA Polymerase (#P505; Vazyme Inc., Nanjing, China) and transfected into the vector pECMV-3 × FLAG-N. Then the TAFAZZIN-WT plasmid was constructed using a ClonExpress® II One Step Cloning Kit (#C215; Vazyme Inc.) for recombination transformation. The constructed plasmid was transfected into human Hek293T cells with 5 *μ*l Lip2000 reagent (Thermo Fisher Scientific Inc., Waltham, MA, USA). The protein (80 μg) was extracted with protein lysate (Beyotime Inc., Shanghai, China), and the protein expression was examined by WB analysis. The results were analyzed using ImageJ software, and it was found that the protein expression of the TAFAZZIN-MUT group was significantly lower than that of the TAFAZZIN-WT group; it was reduced by approximately 31.37% (*p* < 0.001). This indicates that the variation in His104Pro might lead to a decrease in protein expression, thereby affecting protein function (Figure 3). Antibody information: first antibody mouse anti-human FLAG (Cat. #8146; Cell Signaling Technology Inc., Danvers, MA, USA), mouse anti-human *β*-actin (Cat. #3700; Cell Signaling Technology Inc.), dilution ratio 1:1,000; anti-mouse IgG, HRP-linked antibody (Cat. #7076; Cell Signaling Technology Inc.), dilution ratio 1:5,000.

## Discussion

To date, more than 200 patients with BTHS have been reported, most of whom were male, which is related to the X-linked inheritance of the disease (7, 8). Although BTHS has been widely reported, prenatal cases are rare (Table 2). In this study, prenatal ultrasound of a fetus revealed LVNC and ascites at 24^+4^ weeks gestation. After induced labor, fetal skin tissue and peripheral blood samples from the parents were extracted for genetic screening, and it was found that the fetus and its mother carried a novel missense mutation of *TAFAZZIN*, His104Pro. We predicted and analyzed that the mutation could affect protein function through a variety of bioinformatics software models. MD studies showed that the structural stability of the mutant His104Pro was reduced owing to the destruction of the surrounding hydrogen bonds. The WB analysis showed that the His104Pro mutation resulted in decreased protein expression. Through the results of this study, we expanded our knowledge of the mutation spectrum of *TAFAZZIN*; furthermore, we studied the harmful biological implications of the novel His104Pro mutation, demonstrating that it may lead to cardiac dysplasia in the early embryo.

**Table 2:** Characteristics of previously reported prenatal cases of Barth syndrome.

Family	Case	Gestation period	Ascites	Edema	DCM	LVNC	EFE	Pancreas atrophy	Neutropenia	Other	Outcome	Mutation	Ref.
F1	1	31 W	-	+	+	-	+	+	+	NA	Died of pleural effusion and ascites 3 days after birth.	Arg94Gly	(9)
F2	2	32^+^5 W	+	-	-	-	-	-	-	Cardiac enlargement	Death 12 days after birth. Autopsy showed cardiac enlargement. Under the microscope, endocardial fibroelastosis and subendocardial muscle cell vacuolization were suggested. Electron microscopy showed that the mitochondria were enlarged, and the cristae were disordered.	Arg94Gly	(10)
F3	3	30 W	-	-	+	-	-	-	-	NA	Induced labor	Arg94Gly	(11)
F3	4	31 W	+	-	+	NA	NA	+	NA	NA	Drainage of pleural effusion and ascites after cesarean section at 34 weeks, and death on the third day after birth. Autopsy showed renal tubular/cortical and pontosubicular neuronal necrosis.		(11)
F3	5	31 W	-	-	+	-	+	+	-	NA	-		(11)
F4	6	37 W	-	-	+	+	+	NA	+	NA	Stillbirth in cesarean section	c.583 + 5G > A	(11)
F5	7	22 W	-	-	+	-	-	-	-	NA	Delivered at 32 weeks owing to fetal distress and died of ventricular arrhythmia 1 week after birth.	Gly197Arg	(11)
F6	8	NA	-	-	-	-	-	-	-	IUGR	DCM was detected 8 months after birth.	Ile209Asn	(11)
F6	9	33^+4 ^W	-		+	-	-	-	+	IUGR	Unknown	His69Gln	(11)
F7	10	22 W	NA	NA	NA	NA	NA	NA	NA	Multiple malformations	-	Gln280GlyfsX30	(11)
Our study	11	24^+4 ^W	+	+	+	+	+	-	NA	-	Induced labor	His104Pro	Our study

W, weeks; DCM, dilated cardiomyopathy; LVNC, left ventricular non-compaction; EFE, endocardial fibroelastosis; IUGR, intrauterine growth retardation; NA, Not applicable; Ref., reference.

Owing to the poor clinical specificity of BTHS, the diagnosis rate in the fetal period is particularly low. Cardonick et al. (9) first reported a 33-week-old fetus with congenital heart disease, asymmetric intrauterine growth retardation, oligohydramnios, and other phenotypes, with ventricular dysfunction continuing after birth. Finally, the infant was confirmed as having BTHS by 3-MGCA and neutrophil measurements. In a case from 2006, Brady et al. (10) reported a fetus with ascites and cardiac enlargement detected by ultrasound at 32 weeks of gestation. After induction of labor, the embryonic heart tissue was described as having endocardial elastic fiber hyperplasia, endocardial myocardial fiber cavitation, and other pathological features at autopsy. Steward et al. (11) reported several prenatal cases with BTHS family genetic histories in 2010, finding unexplained male fetal edema and serious cardiac structural abnormalities such as DCM and LVNC in ultrasonic diagnosis. According to previous prenatal case reports, BTHS can cause cardiac structural abnormalities of varying severity in the fetal period, and this may be accompanied by intrauterine growth retardation, ascites, and edema. Combined with the ultrasonic characteristics of LVNC and ascites observed in this study, we suggest that when unknown cardiac structural abnormalities such as DCM and LVNC accompanied by ascites or edema are found in prenatal diagnosis, the possibility of BTHS should be considered.

*TAFAZZIN*, also known as G4.5, causes BTHS. It was previously named *TAZ* and is located in the gene-rich region Xq28 (12). TAFAZZIN protein can participate in phospholipid biosynthesis and remodeling of the acyltransferase superfamily. It also plays a key role in CL remodeling. Defective CL has been demonstrated to considerably damage mitochondrial function and structure (13). Many types of *TAFAZZIN* mutations have been reported, including loss of function mutations, such as frameshift and nonsense mutations, and splice and missense mutations. These mutations are distributed throughout the 11 exons of *TAFAZZIN* and can lead to complete protein loss, reduced expression level, or reduced function, such as mitochondrial mistargeting, altered TAFAZZIN macroscopic assembly or folding, and assembly defects (12, 14). In prenatal BTHS cases, including this one, the main *TAFAZZIN* mutation type is a missense mutation, while splicing and frameshift mutations have also been reported sporadically. Noteworthily, most missense variations in prenatal cases are cause pathological changes that affect the binding of protein and substrate. For example, in three cases, the hot spot mutation Arg94Gly (located on the surface of TAFAZZIN) weakens the positive charge of arginine and prevents protein and substrate binding (9–11). Moreover, the His104Pro mutation, identified in this study, and His69Gln and Gly197Arg, previously reported, are located in the buried region. The His69Gln and Gly197Arg mutations have been confirmed to result in protein conformation changes due to altered residue charge, thus affecting substrate binding (11, 14). MD showed that the His104Pro mutation also affected the stability of the protein structure owing to altered charge. WB results revealed that the expression of the mutant protein decreased by approximately 31.37%, indicating that His104Pro decreased protein stability. However, at present, there is not enough evidence supporting that different types or locations of *TAFAZZIN* mutations are related to the severity of BTHS phenotype or prenatal pathological changes (15).

BTHS was once considered simple congenital heart disease; however, increasing numbers of clinical reports have shown that it is a syndrome involving multiple systems. Approximately 70% of children with BTHS show cardiomyopathy, but there are great variations among different individuals. For example, most patients' cardiomyopathy occurs in the form of DCM, LVNC, and endocardial fibroelastosis, while some children can further develop ventricular arrhythmia, heart failure, and even sudden cardiac death owing to myocardial cell damage (16, 17). Neutropenia is the main hematological abnormality index of BTHS patients, which may lead to serious infection. Although cardiomyopathy is the main symptom of this syndrome, some children only show infantile infections rather than heart problems at the first diagnosis (5). The molecular mechanism of BTHS neutrophil deficiency is unclear, but some studies have shown that the stability of respiratory chain complexes is reduced owing to a lack of CL, which increases the reactive oxygen species content and induces phosphatidylserine exposure, resulting in increased neutrophil clearance (18). In addition, skeletal myopathy is also a common BTHS phenotype, usually in the form of non-progressive proximal myasthenia and motor retardation. Children with BTHS generally have a higher rate of protein hydrolysis, which leads to a reduction of skeletal muscle content. The damage to CL remodeling and fatty acid composition caused by *TAFAZZIN* mutation may be one factor contributing to this skeletal myopathy (19). As BTHS may affect the heart and immune system and cause skeletal myopathy, we believe that clinical diagnosis should be combined with multi-system examination.

At present, there is no specific treatment plan for BTHS. Generally, symptomatic treatment is carried out according to the phenotype of the child. Most children with heart failure mainly rely on the use of conventional anti-heart failure drugs including *β*-receptor blockers or angiotensin-converting enzyme inhibitors, while some children may need surgical treatment such as mitral valve replacement or heart transplantation (20, 21). In recent years, research on gene replacement therapy using the adeno-associated virus as a vector has made some progress in a *TAFAZZIN* knockout mouse model. The mice in the experimental treatment group showed increased *TAFAZZIN* expression and restored myocardial and skeletal muscle function (22). Although this technology still requires extensive research using human studies, this animal model has provided strong evidence suggesting it as a potentially effective treatment option for BTHS in humans.

In summary, we report a prenatal case of LVNC with ascites observed during a routine ultrasound examination at 24^+4^ weeks of gestation. Genetic testing of the fetal hemizygotes suggests that a novel *TAFAZZIN* mutation leads to BTHS. This study emphasizes that if cardiac structural abnormalities such as DCM, LVNC, or endocardial fibroelastosis are found in prenatal diagnosis, clinicians should pay attention when a male fetus presents with edema and other phenotypes that may have suggestive value for the further identification of BTHS.

## Data Availability

The datasets presented in this study can be found in online repositories. The names of the repository/repositories and accession number(s) can be found in the article/Supplementary Materials.
